# Emerging Love: A Subjective Exploration of Romantic Bonds in Early Adulthood Within the South Korean Context

**DOI:** 10.3390/bs14121135

**Published:** 2024-11-26

**Authors:** Seo Jung Shin, Ji Seong Yi, Song Yi Lee

**Affiliations:** Department of Counselling and Coaching, Dongguk University-Seoul, 30, Pildong-ro 1 gil, Jung-gu, Seoul 04620, Republic of Korea; tjwjd620822@gmail.com (S.J.S.);

**Keywords:** early adulthood, romantic love, types of love, attachment, adults’ attachment, Q methodology

## Abstract

This study examines and categorises subjective perceptions of love among individuals in their twenties and thirties, offering insights into their viewpoints during early adulthood. The study employed the Q methodology, suitable for analysing subjective perceptions such as perspectives, thoughts, beliefs, and attitudes. It included 23 participants selected through purposive sampling from the 2030 generation residing in South Korea, with 40 statements constructed for the research. The findings revealed four types. Type 1, ‘Love Healing’, experiences psychological well-being through love. Type 2, ‘Love Anxious’, longs for true love but is anxious. Type 3, ‘Love Myself’, expresses hope for healthy love through self-awareness. Type 4, ‘Love Mate’, seeks to maintain psychological love while pursuing independence. This research also explores similarities and differences between existing adult attachment and love types, highlighting the need for practical support tailored to each type. These insights may serve as a foundation for developing coaching and counselling services that help individuals in their twenties and thirties cultivate healthy love and mature into their authentic selves.

## 1. Introduction

Human growth occurs through relationships. It signifies the ability to adapt to society through ongoing interactions with others in various environmental conditions, leading individuals to make choices about their behaviours and lives accordingly. Interactions in human relationships provide psychological stability and self-esteem, which are essential for forming one’s identity [1] and fostering empathy to understand others. While relationships and interactions hold significant meaning at all developmental stages, Erikson [2] suggests that individuals develop their identities by sharing trust and intimacy with others and forming new relationships in early adulthood. If one fails in this process, they may experience feelings of isolation and alienation, which can negatively affect relationships throughout adulthood. Therefore, forming intimate relationships is an important psychosocial developmental task in early adulthood.

In particular, Erikson [2] posited that romantic experiences during early adulthood play a crucial role in developing identity and intimacy. Early adulthood is when individuals establish their sense of identity and form intimate connections through romantic relationships, and the experiences of love during this time significantly impact development throughout adulthood and beyond [3]. The formation of intimacy deepens interpersonal relationships and is an essential process for creating bonds based on trust. One establishes trust through repeated positive interactions and sensitive responsiveness to each other’s needs, serving as a crucial factor in deepening intimacy within relationships [4]. Psychologist Bowlby [5], who explained the importance of emotional bonds with others, proposed the attachment theory, which many scholars have since expanded. Attachment is the process through which a child develops a sense of trust and security, enabling them to explore the world gradually. This sense of emotional safety arises when the primary caregiver consistently responds and provides warm, nurturing care [6]. South Korean society still regards dating and marriage as significant social norms. Consequently, one often associates romantic experiences with an individual’s social accomplishment and successful transition into stable adulthood. In the Korean context, strong social stigmas around failure in dating or marriage may exert additional psychological pressure on individuals. Such societal expectations can influence personal attachment formation and emotional development [7].

These attachment experiences also impact interpersonal relationships in adulthood, with emotional and behavioural patterns in romantic relationships often sharing similar characteristics to those observed in parent–child relationships [8]. This stance implies that children who form stable attachments with their parents are more likely to develop healthy interpersonal relationships as adults. The family-centred culture and emphasis on relational harmony in South Korea reveal unique attachment patterns within the Korean context. In addition to the relationship with the primary caregiver, emotional bonds within Korean society and familial harmony contribute to the formation of secure attachment. Individuals with secure attachment tend to exhibit healthier emotional interactions in relationships with parents, friends, and romantic partners [9]. The attachment formed during adolescence continues to impact adulthood. Adolescent attachment is a crucial predictor of resilience in adulthood, specifically the ability to cope effectively with stress and adversity [10]. Additionally, the type of attachment established during adolescence influences the formation of trust and emotional bonds in adult relationships, playing a significant role in creating predictability in interpersonal interactions [11].

**Figure 1:**
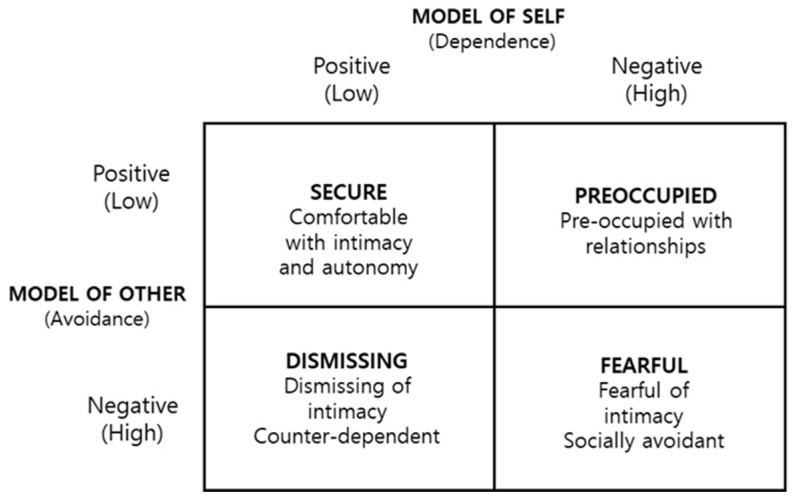
Model of adult attachment [13].

Scholars have provided different categorisations for the concept of adult attachment, which refers to the patterns of child attachment proposed by Bowlby [5] in the context of adult romantic relationships. For example, Hazan and Shaver [12] identified three types of attachment—secure, anxious, and avoidant—while Bartholomew and Horowitz [13] distinguished four types based on the dimensions of self-awareness and other awareness: secure—viewing oneself and others positively and maintaining close relationships; preoccupied—holding a negative self-view and excessively depending on others in relationships; dismissing—evaluating oneself positively but avoiding intimacy due to a lack of trust in others; and fearful—negatively assessing oneself and others, desiring close relationships while simultaneously fearing them (Figure 1) [13]. Thus, the attachment formed during adolescence, similar to the attachment relationships established with primary caregivers in childhood, also influences interpersonal relationships in adulthood.

Recently, the term ‘love and life balance’ (‘러라밸’ in Korean) has gained popularity in Korean society, following the concept of ‘work and life balance’. This new term emphasises the importance of maintaining a balance between love and life, indicating that love should not interfere with one’s daily routine. Amid economic challenges and social changes, South Korea’s millennial generation demonstrates a growing preference for selective and pragmatic relationships that align with individual needs. This generation increasingly forms relationships in a goal-oriented and practical manner, less constrained by traditional social expectations [14]. This phenomenon reflects a social trend that prioritises personal convenience; however, it contrasts with the significant role that interpersonal relationships play in providing emotional support and overall satisfaction in their lives. The stress experienced in interpersonal relationships is closely related to the academic stress of college students in early adulthood, which impacts their overall academic life [15]. Furthermore, higher interpersonal skills positively influence adaptation to college life, and social support greatly assists students in adjusting to new environments [16]. Thus, it is evident that interpersonal relationships are a significant concern during early adulthood and an essential element of overall life satisfaction.

In 2022, the Korea Population, Health, and Welfare Association conducted an online survey involving 1047 unmarried young individuals to assess the status of non-married youth. The survey revealed insights into their dating experiences, non-dating experiences, perceptions of sexuality, and sexual experiences. According to the findings, 65% of young people reported non-dating, with 70% of this group voluntarily non-dating. This generation is beginning to experience a non-dating trend beyond being unmarried [17]. Kim et al. [18] noted an increasing number of individuals who struggle to express their emotions and feel burdened by the overwhelming social pressure to be happy amidst anxiety and concern. This situation has led many to seek emotional proxies as they have nowhere to express their feelings [19]. Thus, they desire to experience romance through media or framed observational entertainment programs instead of through real-life romantic relationships.

Examining love in early adulthood through social phenomena reveals a significant gap between reality and actual desires. The various issues from this disparity affect early adulthood and demonstrate a cyclical interconnectedness with societal problems. Psychoanalyst and social psychologist Fromm [20] posited that contemporary society presents a paradoxical situation where individuals must fulfil the existential task of self-realisation amidst an incessant flow of competition. Fromm argued that modern individuals have become increasingly distant from aspects of the self to the extent that they may forget their identities. This argument has a close link to the realities of South Korean society, reflecting a phenomenon where individuals may lose themselves and experience identity erosion amid intense competition and pressures for achievement. As people strive to meet external expectations in areas like entrance exams and employment, opportunities for self-actualisation diminish, leading to a growing sense of emotional emptiness [21]. He emphasised that understanding love is essential for recovery. Furthermore, he asserted that the only way to realise spontaneity, which is the source of one’s inherent personality and a happy life without sacrificing the self, is through love.

Fromm [20] articulated that love involves connecting with the other by giving everything, including joy, interest, knowledge, sorrow, and even life itself, thereby enriching their existence and allowing oneself to feel alive within that connection. He posited that individuals discover each other’s true selves through such love and evolve into more mature human beings. Hatfield and Sprecher [22] defined love as a combination of human emotions and elements of behaviour, cognition, and affection, reflecting our actions, beliefs, and feelings. Further, existential psychologist May [23] defined love as ‘delight in the presence of the other person and an affirming of his value and development as much as one’s own’ (p. 182), highlighting the importance of acquisitive and compassionate love towards those different from oneself. People in Eastern cultures often express love discreetly and keep it within rather than display it openly to others, in line with cultural tendencies. Historical records across cultures reveal that humans have consistently sought to pursue and preserve love. While expressions of love may vary between Eastern and Western cultures, the fundamental essence of love remains unchanged [24].

Since the 1990s, the study of love has emerged as a significant area of research within social psychology. Sternberg is a prominent scholar who conceptualised and studied love, culminating in the Triangular Theory of Love, which classifies love’s components into intimacy, passion, and commitment. Canadian psychologist John Alan Lee [25] proposed the Colour Wheel Theory of Love, categorising love into six distinct types and outlining the characteristics of each type. Within this framework, the primary categories include eros (passionate love), ludus (playful love), and storge (companionate love), further subdividing into three subgroups: mania (possessive love), agape (selfless love), and pragma (pragmatic love).

Love can be a crucial element in personal growth and transformation, especially in early adulthood. The significance of love becomes even more profound as one matures into adulthood. Erikson [26] posited that experiencing an appropriate balance of isolation and intimacy can facilitate the development of the mature self-capacity known as ‘love’. He further emphasised that romantic experiences of love provide opportunities for individuals to discover new facets of themselves and are vital in uncovering their true identities. Fredrickson [27] argues that romantic love enhances positive emotions, serving as a critical factor in building psychological resources. Similarly, Levinson [28] notes that romantic experiences during early adulthood are fundamental for self-understanding and relationship development, which contribute to forming a mature sense of self.

In summary, forming intimate relationships and experiencing love during early adulthood is necessary for the development of personal identity and intimacy, which, in turn, positively influences self-esteem. Consequently, love is the root of human existence. It is crucial in growth activities aimed at pursuing an ideal self-image and significantly influences the satisfaction or frustration of psychological needs [29].

A review of previous studies on love indicates that researchers have explored love and romantic relationships among university students in South Korea. One narrative study investigated male and female university students’ perceptions of the definition of love, revealing that both groups commonly perceive love as a ‘feeling’ [30]. University students tend to accept love as a desired emotional experience that can make their existence more meaningful. In contrast to this desire for existential meaning through love, recent studies on university students’ views on marriage have shown a significant decline in positive perceptions of marriage [31]. Furthermore, examining the relationship between attitudes towards marriage and types of love indicated that those with positive or evolving values regarding marriage tend to exhibit a strong inclination towards ‘eros’ (passionate love). An in-depth interview study by Oh and Park [32] on university students’ relationships and happiness revealed that pursuing only one type of love can hinder the recognition of happiness. This finding suggests that a diverse range of love types is essential for sustaining healthy and enduring love. Thus, previous research underscores the necessity for a detailed investigation into perceptions of love in early adulthood.

Although studies have used Sternberg’s three components of love [33,34,35], they primarily conducted quantitative research. Choi [36] researched the components of love based on the Enneagram personality types and suggested that love manifests through individuals’ psychological dimensions, which gives rise to various attitudes and types of love. This finding also highlights the need for qualitative research grounded in in-depth approaches. In addition, Choi and Jun [37] emphasised the necessity of qualitative exploration concerning how one perceives love from an essential perspective.

For several years, the study of love as a subjective experience has been a topic of interest in psychology [38]. Studies have partially addressed how individuals perceive love in early adulthood [39,40,41,42]; however, few studies in this field have deeply analysed subjective perceptions of love from diverse perspectives. This lack suggests that psychological inquiry into how individuals uniquely perceive the meaning and experience of love is still evolving. Therefore, this study utilises the Q methodology to categorise and examine the characteristics of subjective perceptions of love among individuals in early adulthood. The findings serve as a foundation for developing coaching and counselling services that facilitate healthy expressions of love and contribute to the mature growth of authentic self-identity in early adulthood.

The research questions (RQs) for this study are as follows:

RQ1. What are the types of perceptions regarding love in early adulthood among South Koreans?

RQ2. What are the characteristics of each perception type regarding love in early adulthood among South Koreans?

## 2. Study Method

### 2.1. Research Procedure

The Q methodology is a research method developed by William Stephenson in the 1930s [43]. In research, generalisation is typically associated with statistical inference based on large samples, assuming that the findings apply to an entire population. However, the Q methodology emphasises subjectivity rather than data quantification and focuses on developing theories [44]. The core of Q methodology lies in exploring the correlations among individuals regarding a specific topic and categorising and interpreting these individuals’ perspectives [45,46]. Generalising the research findings to the entire population is not a concern to Q-methodologists; rather, they focus on identifying individuals’ subjective patterns of thought. The belief is that these patterns exist within the population from which the researcher draws the sample [47]. The Q-sample assumes that such patterns only exist within the group. In other words, ‘When we discover that X has blue eyes, we can argue that there may be others with blue eyes like X, but we cannot claim that everyone else has blue eyes’ [48]. It involves quantifying individuals’ subjective tendencies or values and interpreting the resulting categorised types [49]. Thus, the Q methodology is an objective and practical research approach suitable for understanding the cognitive structures underlying individuals’ subjective perspectives [50].

**Figure 2:**
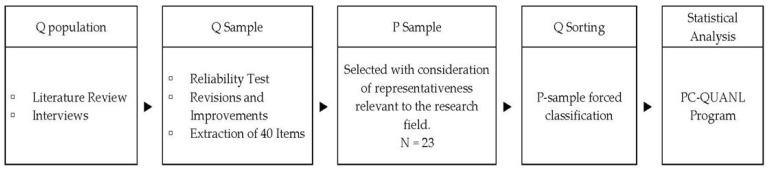
Q methodology steps.

Consequently, this study uses the Q methodology to categorise perceptions of love in early adulthood and discern the meanings associated with each type. The research process involves the following steps: constructing the Q sample, assembling the Q sample from the Q population, selecting the P sample, and classifying the Q sample through the P sample, followed by data analysis. We conducted this study after receiving approval from the Dongguk University Institutional Review Board (IRB no. DUIRB202308-22) following the procedure illustrated in Figure 2.

#### 2.1.1. Organisation of Q Population

In the Q methodology context, the population consists of all possible subjective statements that individuals can express. We can categorise subjective statements as messages, concepts, ideas, or communicable expressions [51]. The Q population refers to a collection of items related to the research topic gathered to study individuals’ subjective characteristics within a culture [46]. In this study, prior to forming the population, we collected a Q population to examine perceptions of love in early adulthood by reviewing 11 academic theses and journal articles with keywords such as ‘perception of love in early adulthood’, ‘love in early adulthood’, and ‘perceptions of love’. The titles are as follows. ‘The Relationship Between Romantic Attachment, Self-Esteem, and Types of Love’ [52], ‘A Study on the Conceptual and Developmental Understanding of Love in Adulthood’ [53], ‘A Study on College Students’ Types of Love and Sexual Attitudes’ [54], ‘The Impact of the Importance of Basic Psychological Needs and Gender Differences on the Components of Love: A Study on Romantic Partners’ [55], ‘An Exploratory Study on the Attitudes Towards Love in Unmarried Adult Males: Focusing on Gender Discriminatory Perceptions and Gender Role Conflicts’ [56], ‘A Study on Theater Therapy for Understanding Love’ [29], ‘Types of Love, Self-Esteem, Trust, and Relationship Satisfaction in Early Adulthood’ [57], ‘A Study on the Elements of Love Perceived by College Students’ [37], ‘Measuring Passionate Love in Intimate Relationships’ [22], ‘Dating Apps: Towards Post-Romantic Love in Digital Societies’ [58], and ‘Love and Relationship Satisfaction as a Function of Romantic Relationship Stages’ [59].

Additionally, we secured 93 statements through internet search engines such as Google, Naver, and Daum. Following this, we conducted semi-structured interviews with four individuals: one male and one female in their twenties, and one male and one female in their thirties. After explaining the purpose of the research and obtaining consent, we conducted the interviews using semi-structured questions, including ‘What comes to mind when you think of love’, ‘What does love mean to you’, ‘What kind of love do you consider to be good love’, ‘Conversely, what kind of love do you think is not good’, ‘What aspects do you think are important regarding love’, ‘What experiences have you had related to love’, and ‘Finally, what else would you like to share about love’? Each interview lasted approximately 50 min, resulting in the addition of 49 statements. This study aimed to broadly explore the subjective perceptions and experiences of love held by early adulthood participants, focusing on their subjective perspectives on experiencing and interpreting love. Thus, we constructed 142 Q population items.

#### 2.1.2. Selection of the Q Sample

The Q sample comprises extracted statements from the Q population, which participants use in Q sorting [46,60]. The Q sample underwent validity verification in this study across three distinct phases. We organised the 142 statements in an Excel file and categorised them based on their content, as follows: psychological state, attitude/method, reality, growth, element, benefit, and self-awareness. Subsequently, we classified the statements as positive, negative, or neutral. Through a thorough review of the organised statements, we modified and deleted statements to address redundancy and ambiguity, resulting in the final selection of 40 statements (Table 1). Following this, one individual in their twenties and one in their thirties assessed the appropriateness and comprehensibility of the statements. Finally, we conducted a pre-test with two experts familiar with the Q methodology. This process culminated in the final selection of 40 statements that effectively represent the Q population and exhibit high discriminative validity for the Q sample.

#### 2.1.3. Composition of P Sample

The P sample refers to the research participants who classify the Q sample [61]. Since the Q methodology does not aim for demographic generalisation, the P sample’s demographic representativeness is unnecessary [62]. Furthermore, the Q methodology addresses intraindividual rather than interindividual differences in significance, allowing for flexibility in the number of participants in the P sample. Consequently, a small sample size is preferred, with an appropriate number of participants being approximately 40 ± 20 [63]. Additionally, selecting participants relevant to the research topic is crucial to enhancing the study’s quality [49]. Therefore, this study utilised purposive sampling to select 23 individuals as the P sample based on the research objectives.

#### 2.1.4. Q Sample Sorting

**Figure 3:**
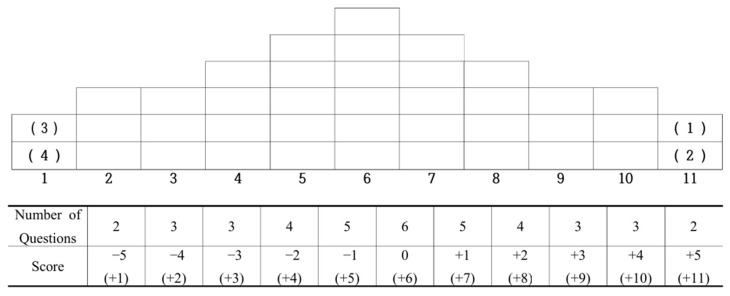
Q sorting distribution chart.

Q sorting refers to how the P sample arranges statements from the Q sample in order of importance based on their perspectives [46]. The Q sorting task differs from commonly used quantitative scales in psychology, such as the Likert scale, as it measures individual preference differences [64]. The method of Q sorting involves participants reading each statement and categorising them according to a forced distribution method [65]. The P sample conducted Q sorting following the Q sorting procedure outlined by Kil et al. [49]; Figure 3 illustrates the Q distribution.

During Q sorting, we instructed research participants to place statements at either end of the spectrum based on their level of agreement, filling from the outer edges towards the centre while organising neutral statements. We guided participants to review the entire set and adjust the items in the Q grid. Finally, we asked them to provide reasons for the two statements with which they most agreed and disagreed. This opportunity allows for a detailed explanation of the participants’ opinions and thoughts, which can be useful for factor interpretation in subsequent data analysis [46]. This study’s P sample conducted Q sorting from 9 to 23 October 2023. To facilitate understanding of the Q sorting, we prepared an instructional document. Among the participants, we engaged seven face-to-face, while 16 participated remotely (via phone or video conference). The Q sorting took approximately 30 to 40 min for each participant, and we provided coffee coupons as a token of appreciation for their participation.

#### 2.1.5. Data Analysis

The present study utilised the QUANL program, which maximises the variance explained for analysis [46]. After confirming the statement numbers of the Q sort distribution, we scored the collected data from 1 to 11 points and coded and entered the weighted values into the computer. Subsequently, we employed the PC-QUANAL program, a Q sorting data analysis tool, utilising principal component factor analysis as the Q factor analysis method. We determined the number of factors based on an eigenvalue threshold of 1.0000 or greater to ensure optimal classification of types. Additionally, we used Q statements with a Z-score greater than one to identify the characteristics of each type. Furthermore, by focusing on participants with high weights within each type, we identified the items with which the participants most agreed and disagreed and documented the reasons, serving as a reference for interpreting the characteristics of each type.

## 3. Study Results

### 3.1. Result Analysis

We categorised the results of the subjective exploratory research on romantic bonds in early adulthood into four types. Table 1 presents the Z-scores for each statement associated with each type, along with their categorisation based on the content of the statements. For the categorisation process, we documented and organized 142 statements collected from the Q population into an Excel file. We then classified these statements into ‘psychological state’, ‘attitude/method’, ‘reality’, ‘growth’, ‘element’, ‘benefit’, and ‘self-awareness’ based on their content. We further classified these statements into positive, negative, and neutral categories and selected the final 40 questions through a process of revision and elimination.

Table 2 summarises the results, revealing four distinct types. The eigenvalues for each type are as follows: Love Healing = 8.8730, Love Anxious = 1.8600, Love Myself = 1.5812, and Independent Love = 1.0124, with a cumulative variance of 0.5787.

The correlations among the four types are in Table 3. The correlation between Love Healing and Love Myself is the highest at 0.614, followed by 0.144 between Love Anxious and Independent Love. Notably, the Q methodology does not assume complete independence among factors but focuses on identifying factors. Therefore, researchers do not debate the significance of the correlation coefficients in the factor extraction process [46].

Among the 23 participants, 11 comprised Type 1 (Love Healing), 3 were Type 2 (Love Anxious), 5 were Type 3 (Love Myself), and 4 were Type 4 (Independent Love). Supplementary Material Table S1 presents the demographic characteristics and factor weights of the four types.

### 3.2. Perception Type Characteristics

#### 3.2.1. Type 1: Love Healing

Love Healing’s characteristic is experiencing psychological well-being through love. The statements with which those in the Love Healing type agreed the most were ‘Having a loving partner helps provide psychological stability’ (Q15, Z = 1.53) and ‘Comfort makes the process of love richer and more stable’ (Q1, Z = 1.51). Conversely, the statements with the highest disagreement were ‘I find that dating through apps is more comfortable than meeting in person’ (Q28, Z = −1.92) and ‘Not giving my heart deeply in a relationship is a way to protect myself’ (Q16, Z = −1.59). Table 1 provides the representative statements and their Z sores for the Love Healing type.

In the Love Healing type, the statement with the highest difference from the average Z-scores of other types was Q19: ‘Love helps me forget the hardships of life’ (Diff. = 2.284). Conversely, the statement with the lowest difference was Q16: ‘Not giving my heart deeply in a relationship is a way to protect myself’ (Diff. = −1.730). The statements from this type that exhibited a Z-score difference of ±1.00 or greater compared to the averages of other types are in Supplementary Materials.

P23, a Love Healing participant, stated:

I feel a sense of stability when I experience comfort, allowing me to reveal my true self. The same goes for my partner. As we accumulate such moments, respect and trust build together, creating a bond I can share with someone for a long time.

Similarly, P3 expressed:

Having spent a long time with my current boyfriend, we have become close enough to discuss things I cannot share with my family or friends. I believe he will always be by my side, and it feels like I have found my closest friend in life.

Thus, those in the Love Healing category reflect positive emotions such as psychological stability, trust, and respect through love. They highlight the characteristics of inner growth through relationships. Based on these findings, we designated those in this category as the ‘Love Healing’ type because they experience psychological well-being through love.

#### 3.2.2. Type 2: Love Anxious

While the Love Anxious type longs for true love, they are anxious about love. The statements with which the Love Anxious type agreed most were ‘I have not experienced true love yet’ (Q12, Z = 2.25) and ‘I am not sure if I will ever meet someone I truly love in my lifetime’ (Q7, Z = 1.49). Conversely, the statements with the lowest levels of agreement were ‘Love is something that people without worries (like employment or money) can pursue’ (Q40, Z = −2.17) and ‘I find that dating through apps is more comfortable than meeting in person’ (Q28, Z = −2.07). Table 1 provides the representative statements and their Z-scores for those in the Love Anxious category.

In the Love Anxious group, the statement with the highest difference in Z-scores compared to the means of other types is Q12, ‘I have not experienced true love yet’ (Diff. = 3.778). Conversely, the statement with the lowest difference is Q9, ‘The degree of love is proportional to mutual trust’ (Diff. = −1.788). The statements from this type that exhibit a Z-score difference of ±1.00 or more compared to the means of other types are in Supplementary Materials.

The participant with the highest factor weight in the Love Anxious category, P22, stated:

Since I have never experienced love, I am unsure what love is or even if it exists. Therefore, I have a strong desire to experience it. Moreover, I ponder what is necessary for love to endure. I believe it involves supporting each other’s growth. The continuity of love is not about appearances or money but rather about encouraging each other’s dreams and helping one another progress through those dreams. I believe that is what sustains love.

P8 expressed:

I think we cannot change people and that they remain the same. There is no perfect person, and I am not perfect either. Therefore, I believe love is about understanding and accepting myself and the other person as we are, which is why I chose this perspective.

In this way, those classified as Love Anxious recognise that healthy relationships involve mutual growth, understanding, and respect for one another, which they perceive as true love. They also exhibit feelings of anxiety regarding forming intimate relationships. Based on these characteristics, we designated those in this group as the ‘Love Anxious’ type because they long for true love, although they are anxious about it.

#### 3.2.3. Type 3: Love Myself

Those in the Love Myself group express hope for healthy love through self-awareness. The statements with which those in the Love Myself category most agreed were ‘I believe that knowing myself well is essential for having good love’ (Q24, Z = 2.43) and ‘When I love, I learn to care for others’ (Q13, Z = 1.73). Conversely, the statements that received the lowest agreement were ‘I find that dating through apps is more comfortable than meeting in person’ (Q28, Z = −1.83) and ‘I have not experienced true love yet’ (Q12, Z = −1.70). Table 1 provides the representative statements and their Z-scores for those in this group.

In the Love Myself group, the statement with the highest difference in Z-score of at least ±1.00 compared to other types is Q16: ‘Not giving my heart deeply in a relationship is a way to protect myself’ (Diff. = 1.978). Conversely, the statement with the lowest difference is Q23: ‘Love and marriage are separate’ (Diff. = −0.213). The statements relating to this type’s Z-score difference of at least ±1.00 compared to other types are in Supplementary Materials.

P7, with the highest factor weight among the Love Myself group, stated:

My partner feels like a mirror to me when I am in love. They bring out aspects of myself I was unaware of, and I sometimes have to confront traits I dislike. I believe that the extent to which I understand myself allows for healthy communication in relationships based on that self-awareness.

Similarly, P4 expressed:

To adjust and coordinate with any partner, I must clearly understand what I like, dislike and my goals. If I change depending on my partner, I might forget who I am at some point; therefore, I believe starting a relationship with a high level of self-awareness and self-esteem is essential.

Thus, those categorized as ‘Love Myself’ reflect a desire to maintain a healthy relationship through self-awareness. Based on these characteristics, we designated this type as ‘Love Myself’ because it expresses hope for healthy love through self-awareness.

#### 3.2.4. Type 4: Independent Love

Those in the Independent Love group seek to maintain psychological love while pursuing independence. The statements with which this type most agreed were, ‘I believe that knowing myself well is essential for having good love’ (Q24, Z = 2.10) and ‘A good romantic relationship seems to involve resolving conflicts in a healthy way’ (Q30, Z = 1.66). Conversely, the statements with the lowest levels of agreement were ‘I find that dating through apps is more comfortable than meeting in person’ (Q28, Z = −2.16) and ‘I have not experienced true love yet’ (Q12, Z = −2.09). Table 1 provides the representative statements and their Z-scores for the Independent Love type.

Among those of the Independent Love type, the statement with the highest Z-score difference of ±1.00 compared to other types is ‘Love and marriage are separate’ (Q23, Diff. = 2.517), while the statement with the lowest Z-score difference is ‘I have not experienced true love yet’ (Q12, Diff. = −1.998). The statements in the Independent Love group that exhibit a Z-score difference of ±1.00 or more compared to the averages of other types are in Supplementary Materials.

P13, with the highest factor weight among those in the Independent Love group, stated:

As I grow older, I often fear repeating past mistakes from previous relationships, even with someone I’ve known for a long time. Developing a new relationship requires careful communication and effort from the start, which can sometimes feel burdensome compared to the enjoyment of advancing the relationship. Consequently, I tend to consider whether the person is capable of understanding and whether we can connect meaningfully before contemplating the progression of our relationship.

In addition, P19 expressed, ‘I believe I am who I am, and the other person is who they are. As we spend more time together and share experiences, respecting each other’s true selves is essential to achieve harmony in the relationship’. Moreover, P21 remarked:

I consider love a relationship based on sacrifice. When two individuals meet, they invest their time, money, emotions, and other resources for love. As the relationship deepens and evolves into genuine love, I feel a profound sense of commitment, akin to risking everything for that person. Therefore, sacrificing for my partner represents true love to me.

Thus, the Independent Love type reflects characteristics of maintaining psychological love while pursuing independence in personal life. Based on these characteristics, we designated Type 4 as the ‘Independent Love’ type because it seeks to maintain psychological love while pursuing independence.

### 3.3. Consensus Items

Consensus items refer to those statements with which all participants agree [66]. Rather than interpreting the characteristics of each type individually, consensus items allow for a comprehensive understanding of the overall traits across types through common statements. We identified 17 consensus items across the types described in Table 4.

## 4. Discussion

This study explored the different types of love perceptions in early adulthood and examined each type’s characteristics using the Q methodology, which is ideal for analysing subjective perceptions. Through Q factor analysis, we identified four types of subjective perceptions regarding love in early adulthood: Type 1 (the ‘Love Healing’ type) experiences psychological well-being through love, Type 2 (the ‘Love Anxious’ type) longs for true love but is anxious about love, Type 3 (the ‘Love Myself’ type) expresses hope for healthy love through self-awareness, and Type 4 (the ‘Independent Love’ type) seeks to maintain psychological love while pursuing independence.

The Love Healing type comprised 11 participants with characteristics of psychological well-being, such as positive emotions, attitudes towards life, and inner growth, which allowed them to accept themselves positively and form trusting, stable relationships with their partners. These characteristics align with Bartholomew and Horowitz’s [13] ‘secure attachment type’, marked by high intimacy, comfort, and autonomy. This type corresponds to the ‘secure’ category of adult attachment styles. Individuals with secure attachment have a well-integrated sense of self-identity and high self-esteem. They also show high intimacy towards and trust in their loved ones, are open and flexible, and do not find self-disclosure difficult [12,13,67,68]. Due to their positive internal working models, these individuals consider being loved to be natural and express high satisfaction in relationships through the comfort and happiness of mutual trust. Additionally, Marrero-Quevedo and colleagues [69] reported a positive relationship between secure attachment and psychological well-being, which resonates with the characteristics of the Love Healing type.

The Love Healing type also reflects similarities with John Alan Lee’s [25] love styles of eros (passionate love) and agape (selfless love). Frazier and Esterly [70] found that higher levels of passionate and selfless love positively correlated with relationship intimacy, commitment, passion, and satisfaction, while passionate love positively affected trust. This view aligns with the findings related to the Love Healing type. Sternberg’s [71,72] Triangular Theory of Love systematically presents love as intimacy, passion, and commitment. Those in the Love Healing category demonstrate all three components, though with a stronger emphasis on intimacy and commitment. Madey and Rodgers [73] also reported that secure attachment relates to intimacy and commitment, a finding consistent with the findings in the Love Healing group.

The additional statements provided by participants in the Love Healing type reveal a shared perception that the key to love lies in ‘acknowledging and accepting each other’s differences while respecting and understanding the partner and oneself’. Participants emphasised the importance of effort in maintaining a loving relationship. For example, P6 said:

As time goes by, there are fewer new things to discover about each other, and we could mistake this as love fading. Therefore, it is important to acknowledge and respect each other and to work on loving the familiar aspects even more.

Similarly, P10 said, ‘I believe that maintaining the feelings of affection requires paying attention to the partner and treating them with care and affection, rather than neglecting them’. This emphasis on sustaining relationships through effort aligns with Bierhoff and Grau’s [74] findings, which show that individuals with secure attachments tend to maintain long-term stable relationships. On the other hand, perceptions of passionate love, particularly the element of physical and sexual attraction highlighted in John Alan Lee’s [25] passionate love and Sternberg’s [71,72] passion component, were not prominent in the Love Healing type. De Munck et al. [75] suggested that the concept of romantic love might vary across cultures, indicating the need to explore whether these findings reflect characteristics of Korean culture.

We found that the Love Anxious type was characterised by a longing for true love accompanied by anxiety. Three participants in the P sample belong to this type. Love Anxious individuals perceived that they had not yet experienced genuine love, viewing a healthy love as one where both partners support each other’s growth and maintain mutual respect. They also expected personal growth through love. However, they demonstrated a negative outlook on whether true love exists or whether they could experience it. Thus, they preferred cohabitation over marriage, a desire to focus more on self-development than love, and a sense of fear or reluctance to form close relationships. These characteristics reflect a longing for true love and anxiety about forming intimate connections, leading to avoidance behaviours.

This finding aligns with the fearful attachment style in Bartholomew and Horowitz’s [13] model, which shows a mix of approach and avoidance tendencies. Hazan and Shaver’s [12] three-category attachment theory originally classified the fearful style under the avoidant attachment style but later distinguished it as a separate category. People with avoidant attachments display similar traits. Bierhoff and Grau [74] noted that individuals with avoidant attachment struggle to accept their partners as they are, fear emotional closeness, and maintain emotional distance, making it difficult to fall in love or experience passionate love. These characteristics align with those of the Love Anxious type.

Unlike the Love Healing type, which aligned with John Alan Lee’s [25] love types, the Love Anxious type did not correspond to any specific love style. However, Frazier and Esterly [70] found that relationships with higher levels of passionate and selfless love exhibited greater intimacy, passion, commitment, and satisfaction, which contrasts with the tendencies of the Love Anxious type. Additionally, previous research has reported that individuals with ambivalent or avoidant attachment styles display possessive love, characterised by strong jealousy and attachment [76], a trait not observed in the Love Anxious type.

Levy and Davis [77] reported an association between lower levels of anxiety and avoidant with higher levels of intimacy, passion, and commitment, as outlined in Sternberg’s Triangular Theory of Love. In contrast, those in the Love Anxious group demonstrated low recognition of all three components. Supporting statements from Love Anxious participants include ‘I have never experienced love, so I don’t know what love is or whether it even exists’ (P22), ‘Though breakups are sad, I have never been in a relationship that made me want to fall in love again’ (P10), and ‘I don’t want to spend the emotional energy required to meet new people, stay in touch, and think about them’ (P8). However, positive experiences in forming stable relationships can transform attachment styles [78]. Therefore, providing educational and coaching programs to help individuals classified as Love Anxious develop their attachment style and experience fulfilling love relationships may be beneficial.

The Love Myself type exhibited characteristics associated with intimacy and commitment from Sternberg’s [71,72] Triangular Theory of Love. Specifically, Love Myself individuals displayed a commitment to love through self-awareness, leading to a sense of connectedness in intimate relationships and a drive to sustain that love. One of the most notable features of those classified as Love Myself types is how individuals gain self-awareness through their relationship dynamics and their partners’ reactions. Supporting statements from participants in this group include ‘My partner feels like a mirror to me’ (P7), ‘I believe that knowing myself well helps me maintain balance and not be swayed, regardless of who I’m with’ (P4), and ‘Knowing myself well reduces the chances of projecting my dissatisfaction onto my partner’ (P16). This result aligns with Cooley’s [79] concept of the ‘Looking-Glass Self’, which emphasises that social interaction forms and continuously reshapes self-identity. This concept mirrors the Love Myself type’s characteristic of recognising oneself through close, influential relationships with their partner.

Research shows that individuals in early adulthood (twenties to thirties) with a clearer self-perception, including subjective evaluations of their worth or competence, are better equipped for interpersonal competence [80]. Moreover, emotional clarity is essential in conveying one’s needs, intentions, and desires to others while also recognising and responding to the intentions of others in interpersonal relationships [81]. Therefore, for individuals in the Love Myself group, who prioritise understanding their emotions and identifying their needs, coaching programs that enhance self-awareness and communication training for conflict resolution are necessary to foster healthy dialogue and relationship development.

The Independent Love type, the love mate type, seeks to maintain psychological love while pursuing independence in life. This type comprised four participants from the P sample. Although the Independent Love type shared the same statements of agreement and disagreement as those in the Love Myself group, the reasons behind their choices reveal significant differences. In those classified as Love Myself, self-awareness was a way to protect themselves in relationships, promoting a desire for healthy love. In contrast, those categorised in the Independent Love group demonstrated a focus on self over a partner—for example, ‘If I keep adjusting to my partner, I will lose myself and adopt an unhealthy mindset’ (P9), ‘I am myself, and my partner is their self. I believe that relationships are built by sharing moments and building memories together, but it’s important to respect each other’s individuality. I am the main character in my life’ (P19), and ‘One of my priorities is knowing myself. I believe love is a relationship that requires sacrifice, but I see sacrificing for my partner as a sign of true love’ (P21). These statements reflect self-awareness that centres on the individual rather than the partner.

Furthermore, while they perceive their romantic partner as special in their relationships, unlike friends, they recognise that deepening love often brings a sense of security but also requires sacrifices in one’s life. Like those of the Love Myself type, individuals in the Independent Love group combine intimacy, which enhances emotional connection and social support, with commitment, which involves a willingness to sustain love through personal sacrifices [71,72]. However, unlike the Love Myself type, those in the Independent Love group emphasise that love requires sacrifice and commitment, but only if they can maintain their independence. Once they achieve psychological and social independence, they can commit to love, allowing them to form intimacy.

In this regard, the Independent Love type contrasts with the Love Myself type in that they do not necessarily see marriage as the ultimate goal of true love. While they find stability through deep relationships, they also desire mutual respect for their personal lives before reaching that point. This perspective aligns with previous studies on Korean youth’s pursuit of independence, particularly in response to changing societal values and the instability of their personal and macro-level social environments [82,83,84,85]. Thus, the Independent Love type represents a mix of secure attachment, characterised by comfort in relationships and healthy conflict resolution, and dismissing attachment, marked by high self-esteem and strong independence [13]. In diagrammatic terms, we could position this type closer to the secure end of the attachment spectrum while retaining elements of dismissing attachment.

The consensus items included five statements with a standard score of ±1 or higher across all types: ‘A good romantic relationship seems to involve resolving conflicts in a healthy way’ (Q30, z = 1.15), ‘Love is about respecting myself and my partner just as we are (Q32, z = 1.11)’, ‘Balancing work and a romantic relationship feels overwhelming’ (Q37, z = −1.08), ‘Love is something that people without worries (like employment or money) can pursue’ (Q40, z = −1.68), and ‘I find that dating through apps is more comfortable than meeting in person’ (Q28, z = −1.99).

A common feature across the four types is the emphasis on resolving conflicts in a healthy manner and respecting oneself and one’s partner, suggesting that these individuals are contemplating the nature of true love. Lloyd [86] argued for the importance of resolving conflicts based on research findings that healthy conflict resolution leads to positive changes in relationship satisfaction for couples. Furthermore, rather than external conditions (such as employment, work life, or money) serving as negative factors in love, the findings suggest that love can help individuals overcome external difficulties.

This study identified the perception types of love in early adulthood and showed that young adults commonly grapple with the concept of true love and exhibit a consistent desire to improve for the sake of maintaining healthy relationships. Kang [87] revealed that young adults in South Korean society seek love and happiness despite various conflicts, striving to bridge the gap between romantic ideals and reality and pursuing self-reflective love through relationships with others. This viewpoint aligns with the findings of the present study. Despite these commonalities, it was evident that their perceptions of love varied significantly. Rather than categorising them into distinct adult attachment types, the findings revealed that while individuals exhibited characteristics from various attachment types, they more prominently expressed certain attachment styles.

## 5. Conclusions

**Figure 4:**
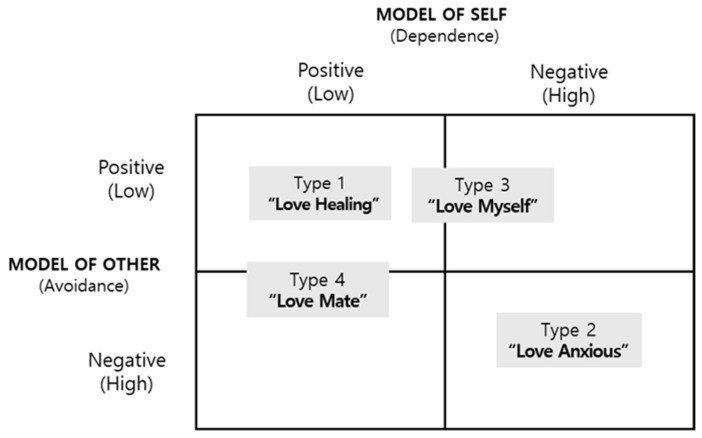
Love perception type model of early adulthood.

This study analysed subjective perceptions of love in early adulthood within South Korean society using the Q methodology, identifying distinct perception types. The significance of this research lies in its pioneering use of Q methodology to classify perception types regarding love in early adulthood. Figure 4 illustrates a schematic representation of this study’s four findings.

The study results indicated that the four perception types relate to Bartholomew and Horowitz’s [13] adult attachment styles, revealing specific similarities and differences. The Love Healing type closely resembled the secure attachment style, while the Love Anxious type exhibited characteristics highly similar to the fearful style. The Love Myself type displayed traits of the preoccupied style, leaning towards the secure attachment style. Finally, the Independent Love type demonstrated features of the dismissing style, which is close to secure attachment. Additionally, we found the four perception types associated with John Alan Lee’s six styles of love and Sternberg’s triangular theory of love. The study also identified the key characteristics of each type.

The Love Healing type experienced psychological stability and positive emotions through love and sought inner growth via romantic relationships. In contrast, the Love Anxious type showed a yearning for true love, with a focus on mutual growth and a tendency towards anxiety. The third type, Love Myself, valued self-awareness and self-esteem as central to relationships, emphasising independence while pursuing mature and healthy love. Finally, the Independent Love type prioritised independence while maintaining emotional bonds, viewing love and marriage separately and valuing a balance between love and personal life. Kang [87] found no direct transfer of Western concepts of romantic love to South Korean society, even those that had evolved with distinct characteristics. He observed that young adults in Korea, facing economic difficulties and rapid social changes, tend to prefer more practical romantic relationships, blending ideals of romantic love with the reality of insecure, individualistic love. This perspective aligns with the characteristics of the Love Anxious and Independent Love types in the present study.

On the other hand, a commonality among all four types was the emphasis on psychological stability and growth through love. Participants shared a commitment to mutual understanding and respect, recognising the importance of relationships, a willingness to resolve conflicts in a healthy way, and the view that love is not a fleeting emotion but a significant element requiring continuous effort. These findings reflect South Korea’s unique social norms, family-centredness, and emphasis on psychological stability, exhibiting a pattern distinct from that of Western societies. Confucianism from China has profoundly influenced South Korea, emphasising relational harmony, still viewing love and marriage as significant social tasks, and reinforcing traditional family structures [88]. In contrast, Western societies emphasise individual independence and self-realisation, placing greater importance on personal choice in love and marriage. Karandashev [89] notes that Western cultures prioritise protecting and maintaining the self-concept, while Eastern cultures, which emphasise interdependence, tend to use preventive strategies to sustain relationships. This tendency impacts romantic relationships, with Eastern cultures showing a correlation between individual change and relationship quality, a connection less relevant in Western contexts.

This study shows how individuals in early adulthood subjectively perceive love within the cultural context of South Korea, highlighting love’s significant role in shaping self-identity and forming mature relationships. These findings may serve as foundational data for developing counselling and coaching services aimed at helping young adults in Korea experience positive and fulfilling love tailored to the characteristics of each identified type. Based on this study’s findings, developing an enhanced love scale that incorporates existing love types and attachment styles for use in universities and field settings for young adults may be valuable in supporting personal growth through love. Finally, future research on the subjectivity of love during the transition to middle adulthood, based on this study, will deepen our understanding of how developmental processes and life transitions shape perceptions of love and attachment types. We expect this will be an expanded study offering profound insights into love during adult development.

## 6. Limitations

The limitations of this study and suggestions for future research are as follows. First, this study employed the Q methodology to examine the subjective perceptions of a small number of participants. While such a small sample size limits statistical generalizability, the Q methodology is designed to categorize subjective perceptions. Therefore, it was suitable for identifying perception types and characteristics regarding love in early adulthood. However, the findings of this study cannot be generalized. Future research should comprehensively investigate the factors influencing love in early adulthood, using quantitative methods to demonstrate how love facilitates personal growth. This would allow for a broader generalization of the findings.

Second, there is a limitation regarding whether the typological characteristics derived from Q methodology adequately reflect subjective perceptions of love. Therefore, we suggest qualitative research, such as interpretative phenomenological analysis, to explore each type’s deeper internal perceptions and experiences regarding love in early adulthood.

Third, this study focuses on exploring perceptions of love within a specific age group in early adulthood. However, experiences of love may evolve as individuals between the ages of 20 and 40 encounter various life experiences and developmental tasks. Therefore, further research could help us understand how perceptions of love differ across ages. A comparative study across age groups, analysing differences in perceptions of love from ages 20 to 40, would help address this limitation. Such research could contribute to understanding the impact of romantic experiences, marital status, and changes in social roles on perceptions of love.

Fourth, this study did not adequately account for potential differences in perceptions of love based on gender. Despite possible variations in expectations and experiences of love by gender, the sample composition and analysis in this study did not allow for a clear explanation of these differences. Future research should explore perception differences by gender in greater detail.

Finally, this study focused on a sample restricted to South Korea, which does not fully account for cultural diversity. As perceptions and experiences of love may vary across cultural contexts, further research should explore love perceptions in other cultural settings. Such studies would enable an evaluation of the cultural universality of the findings and provide a broader understanding of perceptions of love.

## Figures and Tables

**Table 1:** Q statements and Z-scores of love types.

No	Q Statement	Love Healing Z-Scores	Love Anxious Z-Scores	Love Myself Z-Scores	Independent Love Z-Scores	Category
1	Comfort makes the process of love richer and more stable.	1.5	0.8	0.9	0.7	Psychological State
2	Though parting after love is sad, love is something you want to experience again.	0.5	−0.6	0.6	−0.5	Psychological State
3	I often feel fear or annoyance at the thought of forming relationships with others.	−0.1	0.9	−0.6	0.1	Psychological State
4	When I love, I feel jealousy.	0.1	0.6	0.3	−0.3	Psychological State
5	It seems that sharing everyday life with the one I love is happiness.	1.2	−0.3	0.5	0.9	Attitude/Method
6	Seeing my parents makes me not want to get married.	−1.4	−1.1	−0.5	−0.3	Reality
7	I am not sure if I will ever meet someone I truly love in my lifetime.	−1.3	1.5	−0.5	−2.0	Psychological State
8	Romance is important, but I want to focus more on my career and personal growth.	−0.8	0.7	0	0.7	Attitude/Method
9	The depth of love is proportional to the trust between partners.	1.5	−0.6	0.5	1.6	Psychological State
10	Pursuing shared hobbies or goals can strengthen the bond between partners.	0.8	0.9	−0.1	0.6	Attitude/Method
11	I want to have a healthy love, but I am not sure how to do it.	−0.7	1.4	−0.8	−1.3	Attitude/Method
12	I have not experienced true love yet.	−0.8	2.2	−1.7	−2.1	Attitude/Method
13	When I love, I learn the ability to care for others.	0.8	−0.3	1.7	0.4	Growth
14	I believe that helping each other grow is a sign of healthy love.	0.8	1.2	0.7	0.8	Growth
15	Having a loving partner helps provide psychological stability.	1.5	−0.2	−0.2	1.2	Psychological State
16	Not giving my heart deeply in a relationship is a way to protect myself.	−1.6	−0.5	1.2	−0.3	Psychological State
17	Love tends to create a sense of identification between each other.	−0.5	−1.2	−1.0	−0.3	Psychological State
18	I believe loving someone means seeing them as they are, without conditions.	0.6	0.1	−0.3	−0.7	Element
19	Love helps me forget the hardships of life.	1.2	−1.7	−0.6	−1.0	Benefit
20	Love is about gaining my closest friend.	1.3	0.5	1.3	0.1	Benefit
21	Good love is about overcoming challenging times together.	0.7	0.3	−0.2	0.6	Element
22	Love is the most important aspect of human relationships.	0.6	−0.4	−0.1	0.4	Attitude/Method
23	Love and marriage are separate.	−1.1	−1.0	−1.4	1.4	Attitude/Method
24	I believe that knowing myself well is essential for having good love.	0.9	0.3	2.4	2.1	Self-Awareness
25	If I do not like who I am when I am in love, I do not think it’s a healthy love.	0	−0.4	1.4	−0.3	Self-Awareness
26	I feel reluctant to date someone who has experienced many romantic relationships.	−1.1	−0.9	−0.7	0.1	Attitude/Method
27	I prefer living with the person I love rather than getting married.	−1.5	0.8	−1.2	−0.3	Attitude/Method
28	I find that dating through apps is more comfortable than meeting in person.	−1.9	−2.1	−1.8	−2.2	Attitude/Method
29	A satisfying sex life is one of the important aspects of a romantic relationship.	0.2	0.7	0.3	0.7	Element
30	A good romantic relationship seems to involve resolving conflicts in a healthy way.	0.7	0.7	1.6	1.7	Attitude/Method
31	If I like who I am when I am in love, it is a good love.	0.5	1.2	0.9	−0.6	Self-Awareness
32	Love is about respecting myself and my partner just as we are.	1.2	1.1	1.2	1.0	Attitude/Method
33	Sometimes, love is expressed through self-sacrifice.	−0.1	−0.2	−0.3	0.4	Attitude/Method
34	Having more experience in dating can be helpful when in a relationship.	−0.5	−0.4	0.3	−0.1	Attitude/Method
35	At first, relationships may be based on emotional attraction, but later, continuous effort is needed.	0.8	0.2	0.1	0.2	Attitude/Method
36	It seems that who I fall in love with can change who I am.	−0.1	−0.2	0.2	−0.4	Self-Awareness
37	Balancing work and a romantic relationship feels overwhelming.	−0.9	−1.0	−1.6	−0.9	Reality
38	I want to focus more on self-development than on love.	−0.6	0.7	−0.9	0.2	Reality
39	It is sad to have to worry about money while in a relationship.	−1.0	−1.5	−0.3	−1.0	Reality
40	Love is something that people without worries (like employment or money) can pursue.	−1.5	−2.2	−1.5	−1.6	Reality

**Table 2:** Eigenvalues and explanatory variances in the sorting of four love types.

Content	Love Healing	Love Anxious	Love Myself	Independent Love
Chosen eigenvalues	8.8730	1.8600	1.5648	1.0124
Total variance	0.3858	0.0809	0.0680	0.0440
Cumulative	0.3858	0.4667	0.5347	0.5787
Solution variance	0.6666	0.1397	0.1176	0.0761
Cumulative	0.6666	0.0864	0.9239	1.0000

**Table 3:** Correlation coefficients between love types.

Type	Love Healing	Love Anxious	Love Myself	Independent Love
Love Healing	1.000	0.252	0.614	0.598
Love Anxious		1.000	0.248	0.144
Love Myself			1.000	0.573
Independent Love				1.000

**Table 4:** Consensus love items.

No	Statement	Z-Score
30	A good romantic relationship seems to involve resolving conflicts in a healthy way.	1.15
32	Love is about respecting myself and my partner just as we are.	1.11
1	Comfort makes the process of love richer and more stable.	0.98
14	I believe that helping each other grow is a sign of healthy love.	0.88
10	Pursuing shared hobbies or goals can strengthen the bond between partners.	0.53
29	A satisfying sex life is one of the important aspects of a romantic relationship.	0.48
21	Good love is about overcoming challenging times together.	0.36
35	At first, relationships may be based on emotional attraction, but later, continuous effort is needed.	0.32
4	When I love, I feel jealousy.	0.17
22	Love is the most important aspect of human relationships.	0.13
33	Sometimes, love is expressed through self-sacrifice.	−0.05
36	It seems that who I fall in love with can change who I am.	−0.13
34	Having more experience in dating can be helpful when in a relationship.	−0.16
17	Love tends to create a sense of identification between each other.	−0.75
37	Balancing work and a romantic relationship feels overwhelming.	−1.08
40	Love is something that people without worries (like employment or money) can pursue.	−1.68
28	I find that dating through apps is more comfortable than meeting in person.	−1.99

## Data Availability

The datasets generated and/or analysed during the current study are not publicly available to preserve the anonymity of the respondents but are available from the corresponding author upon reasonable request.

## Supplementary Materials

The following supporting information can be downloaded at: https://www.mdpi.com/article/10.3390/bs14121135/s1. Table S1. P Samples and Factor Weights by Love Type. Table S2. Statements of the Love Healing Type with a Z-Score Difference of at Least ±1.00 Compared to the Averages of Other Love Types. Table S3. Statements of the Love Anxious Type with a Z-Score Difference of at Least ±1.00 Compared to the Averages of Other Love Types. Table S4. Statements of the Love Myself Type with a Z-Score Difference of at Least ±1.00 Compared to the Averages of Other Love Types. Table S5. Statements of the Independent Love Type with a Z−Score Difference of at Least ±1.00 Compared to the Averages of Other Love Types.
