# Supplementary material for: Emerging Love: A Subjective Exploration of Romantic Bonds in Early Adulthood Within the South Korean Context
Source: Behav Sci (Basel). 2024 Nov 26;14(12):1135. doi: 10.3390/bs14121135 (PMC11673273; doi:10.3390/bs14121135)
Supplement: Supplementary file 1 [file behavsci-14-01135-s001.zip › behavsci-3257107-supplementary.pdf]

Supplementary Material Table S1. P Samples and Factor Weights by Love Type.

Type	No.	Factor Loading	Gender	Age	Residence	Occupation	Dating Frequency
1 (N = 11)	P1	1.1020	M	29	Seoul	Office worker	3 times
	P2	1.3776	F	29	Seoul	Children's clothing designer	1 time
	P3	1.3229	F	25	Seoul	Freelancer	1 time
	P5	.9120	M	31	Seoul	Unemployed	5 times
	P6	.8661	M	26	Seoul	Business	3 times
	P11	1.2159	M	19	Seoul	College student	1 time
	P14	.8900	F	30	Gyeonggi	Social worker	1 time
	P15	1.1530	F	24	Seoul	Designer	Not disclosed
	P18	.9087	M	29	Seoul	Graduate student	5 times
	P20	1.0547	M	35	Gyeonggi	Social worker	Not disclosed
	P23	1.3675	F	29	Gyeonggi	Unemployed	7 times
2 (N = 3)	P8	.7657	F	28	Seoul	Office worker	Not disclosed
	P10	1.2579	F	29	Seoul	Makeup artist	4 times (adolescence)
	P22	1.3068	F	36	Seoul	Researcher	Not disclosed
3 (N = 5)	P4	.6033	F	26	Seoul	Unemployed	5 times
	P7	1.4535	F	29	Seoul	Graduate student	4 times
	P12	1.4391	F	27	Seoul	Office worker	Not disclosed
	P16	.5349	F	29	Seoul	Clinical trial monitor	7 times
	P17	1.1447	M	35	Gyeonggi	Social worker	Not disclosed
4 (N = 4)	P9	.6623	F	29	Seoul	Office worker	2 times
	P13	1.4012	F	37	Gyeonggi	Graduate student	Not disclosed
	P19	.6866	F	27	Jeju	Office worker	4 times
	P21	.7929	M	22	Gyeonggi	College student	1 time

Supplementary Material Table S2. Statements of the Love Healing Type with a Z-Score Difference of at Least ±1.00 Compared to the Averages of Other Love Types.

No.	Statement	Z-Score	Average Z	Diff.
19	Love helps me forget the hardships of life.	1.198	−1.086	2.284
15	Having a loving partner helps provide psychological stability.	1.533	.262	1.271
7	I am not sure if I will ever meet someone I truly love in my lifetime.	−1.512	−.236	−1.276
8	Romance is important, but I want to focus more on my career and personal growth.	−.801	.506	−1.307
16	Not giving my heart deeply in a relationship is a way to protect myself.	−1.589	.141	−1.730

Supplementary Material Table S3. Statements of the Love Anxious Type with a Z-Score Difference of at Least ±1.00 Compared to the Averages of Other Love Types.

No.	Statement	Z-Score	Average Z	Diff.
12	I have not experienced true love yet.	2.245	−1.532	3.778
7	I am not sure if I will ever meet someone I truly love in my lifetime.	1.489	−1.259	2.747
11	I want to have a healthy love, but I am not sure how to do it.	1.374	−.935	2.309
27	I prefer living with the person I love rather than getting married.	.770	−.997	1.766
38	I want to focus more on self-development than on love.	.711	−.434	1.145
3	I often feel fear or annoyance at the thought of forming relationships with others.	.921	−.203	1.124
5	It seems that sharing everyday life with the one I love is happiness.	−.311	.874	−1.185
13	When I love, I learn the ability to care for others.	−.279	.997	−1.277
24	I believe that knowing myself well is essential for having good love.	.300	1.816	−1.517
19	Love helps me forget the hardships of life.	−1.736	−.107	−1.629
9	The depth of love is proportional to the trust between partners.	−.578	1.210	−1.788

Supplementary Material Table S4. Statements of the Love Myself Type with a Z-Score Difference of at Least ±1.00 Compared to the Averages of Other Love Types.

No.	Statement	Z-Score	Average Z	Diff.
16	Not giving my heart deeply in a relationship is a way to protect myself.	1.192	−.786	1.978
25	If I do not like who I am when I am in love, I do not think it's a healthy love.	1.440	−.224	1.664
13	When I love, I learn the ability to care for others.	1.728	.328	1.400
24	I believe that knowing myself well is essential for having good love.	2.426	1.108	1.318
38	I want to focus more on self-development than on love.	−.900	.103	−1.002
15	Having a loving partner helps provide psychological stability.	−.190	.837	−1.027
23	Love and marriage are separate.	−1.425	−.213	−1.213

Supplementary Material Table S5. Statements of the Independent Love Type with a Z−Score Difference of at Least ±1.00 Compared to the Averages of Other Love Types.

No.	Statement	Z−Score	Average Z	Diff.
23	Love and marriage are separate.	1.372	−1.145	2.517
9	The depth of love is proportional to the trust between partners.	1.628	.474	1.154
11	I want to have a healthy love, but I am not sure how to do it.	−1.309	−.041	−1.267
31	If I like who I am when I am in love, it is a good love.	−.581	.833	−1.414
7	I am not sure if I will ever meet someone I truly love in my lifetime.	−2.001	−.095	−1.905
12	I have not experienced true love yet.	−2.086	−.088	−1.998
